# Promoting well-being in the face of a pandemic: the role of sense of coherence and ego-resilience in the relationship between psychological distress and life satisfaction

**DOI:** 10.1177/00812463221113671

**Published:** 2022-08-02

**Authors:** Anita Padmanabhanunni, Tyrone B Pretorius

**Affiliations:** Department of Psychology, University of the Western Cape, South Africa

**Keywords:** COVID-19, depression, ego-resilience, hopelessness, loneliness, sense of coherence, South Africa

## Abstract

COVID-19 has impacted negatively on the lives and academic activities of university students. This has contributed to increasing levels of psychological distress among this population group. Intrinsic and contextual factors can mediate the psychological impact of the pandemic. The study focuses on sense of coherence and ego-resilience as potential protective factors on indices of psychological distress and life satisfaction. Participants were undergraduate students (*N* = 337) at a South African university who completed six self-report questionnaires, namely, the Beck Hopelessness Scale, the University of California Los Angeles Loneliness Scale, the Center for Epidemiological Studies Depression Scale, the Sense of Coherence Scale, the Ego-Resilience Scale, and the Satisfaction with Life Scale. To examine the direct and mediating effects of sense of coherence and ego-resilience on psychological distress, structural equation modeling was used. Compared to previous research, greater psychological distress was found in the current sample. Moreover, while the hopelessness–life satisfaction relationship was only partially mediated by protective factors, the depression–life satisfaction relationship was fully mediated by sense of coherence and ego-resilience. The direct association between ego-resilience as well as sense of coherence and life satisfaction was significant, suggesting that these factors have a health-sustaining role.

There is a rapidly growing body of research suggesting there is an association between the COVID-19 pandemic and indices of psychological distress and well-being (Serafini et al., 2020; Son et al., 2020). University students have been differentially impacted by the pandemic and its related prevention measures (Wang et al., 2020; Zhai & Du, 2020). Even prior to the pandemic, university students were identified as being at risk for anxiety, depression, and suicidality, among other negative indices of psychological well-being (Zhai & Du, 2020). The pandemic prompted the closure of many universities globally and necessitated a transition to distance learning (Kecojevic et al., 2020). In South Africa, the government instated a strict lockdown in March 2020 in response to the outbreak of COVID-19. This lockdown entailed restrictions on in-person socializing, curfews enforced by the police and military, work-from-home directives, travel restrictions, and closure of all educational institutions (South African Government Gazette, 2020).

Higher-education institutions had to suspend their academic calendar and rapidly transition from face-to-face to electronic mediums of instruction. University students were required to leave their campuses and those living in residences had to return to their families and stay confined at their homes under the national lockdown regulations (South African Government Gazette, 2020). To salvage the academic year, universities rapidly transitioned to online modes of learning and teaching (Padmanabhanunni & Pretorius, 2021). However, this action sparked a debate regarding the extent to which these shifts considered the needs of a significant majority of students who were socioeconomically disadvantaged (Naidoo & Cartwright, 2020).

Generally, effective engagement with online learning requires access to digital technology resources and Internet connectivity, which many students in South Africa lack (Padmanabhanunni & Pretorius, 2021). Moreover, the challenges associated with remote learning are increasingly pronounced for students from disadvantaged backgrounds, who reside in impoverished communities (Naidoo & Cartwright, 2020). These students have to also contend with living in homes characterized by poor infrastructure, overcrowding, limited material resources, food insecurity, and limited privacy and physical space to engage with their studies (Naidoo & Cartwright, 2020).

International research has confirmed that COVID-19 had a disruptive effect on the daily lives and educational routines of university students, which has contributed to increased levels of psychological distress, including high levels of anxiety, loneliness, depression, and hopelessness (Cao et al., 2020; Wang et al., 2020). Some studies have also shown that certain contextual and intrinsic characteristics can play a role in mediating the psychological impact of stressful events, including willingness to engage with social support networks (Brown et al., 2020), self-esteem (Kong et al., 2013), sense of coherence (Gómez-Salgado et al., 2020), locus of control (Goldzweig et al., 2016), and fortitude (Padmanabhanunni & Pretorius, 2020).

This study characterizes the mental health consequences associated with COVID-19 among South African university students and investigates the potential protective role of ego-resilience and sense of coherence with respect to indices of psychological distress and life satisfaction. Ego-resilience and sense of coherence represent the personality characteristics that can play a role in mediating the impact of stressful life events (Gómez-Salgado et al., 2020). Sense of coherence refers to the ability to perceive stressful life events as understandable, meaningful, and manageable events, whereas ego-resilience refers to the capacity to negotiate internal and external stressors in a flexible and resourceful way (Block & Kremen, 1996). Both sense of coherence and ego-resilience have been positively correlated with satisfaction with life and psychological well-being (e.g., Gómez-Salgado et al., 2020; Y. H. Kim & Koh, 2016).

## Method

### Participants

A cross-sectional survey design was used in this study. Participants were randomly sampled with a 6% confidence interval and 95% confidence level. The sample comprised undergraduate students (*N* = 337) enrolled at a South African university. The majority of the sample were women (77.2%), between the ages of 16 and 28 years with a mean age of 21.95 (±4.7) years. The higher proportion of women is consistent with the University’s enrollment data which reflects that a greater proportion of women than men are enrolled at the undergraduate level.

### Instruments

Participants completed six self-report questionnaires: The University of California Los Angeles Loneliness Scale (UCLA-LS; Russell et al., 1980), the Beck Hopelessness Scale (BHS; Beck et al., 1974), the Center for Epidemiological Studies Depression Scale (CES-D; Radloff, 1977), the Satisfaction with Life Scale (SWLS; Diener et al., 1985), the Sense of Coherence Scale (SOC-13; Antonovsky, 1987), and the Ego-Resilience Scale (ER89; Block & Kremen, 1996).

The UCLA-LS consists of 20-items that assesses subjective feelings of social isolation and loneliness. Respondents rate their experiences on a 4-point Likert-type scale that ranges from *Often* to *Rarely*. In this scale, “How often do you feel that you lack companionship?” and “How often do you feel left out?” are the examples of the scale items included. Research (e.g., Doğan et al., 2011) has demonstrated that the UCLA-LS has satisfactory reliabilities (α ranging from .94 to .96). Pretorius (1993) reported an α coefficient of .77 in a South African sample.

The SWLS is a 5-item measure of satisfaction with life, including “In most ways my life is close to my ideal” and “I am satisfied with my life.” The response format is a 7-point scale that ranges from *Strongly disagree* (1) to *Strongly agree* (7). Satisfactory reliability coefficients (α = .91; Lorenzo-Seva et al., 2019) have been reported for this scale. In South Africa, Koen et al. (2022) reported an α coefficient of .79 for a Setswana version of the SWLS, while Schutte et al. (2021) confirmed the unidimensional structure of the scale when used with a South African sample.

The BHS is a 20-item measure of hopelessness where responses are dichotomized as true/false. Examples of items include “I don’t expect to get what I really want” and “My future seems dark to me.” For this instrument, Beck et al. (1974) reported a satisfactory reliability coefficient of .93. The measure was also validated against a clinical assessment of hopelessness, and a validity coefficient of .74 was reported (Beck et al. 1974). Heppner et al. (2002) reported a reliability coefficient of .82 for the BHS when used with Black South African students.

The CES-D comprises of 20 items and is used to screen for depression. It consists of seven items that focus on somatic symptoms, seven items that focus on depressed affect, four items that focus on positive affect, as well as two items that focus on interpersonal problems. Responses range from *Rarely or None of the time* to *All of the time*. The reliability coefficients for this scale have been reported to be satisfactory (α = .70–.90; González et al., 2017). In South Africa, the CES-D also demonstrated satisfactory reliability and validity as well as a similar factor structure to previously reported findings (Pretorius, 1991).

The Ego-Resilience Scale (ERS) consists of 14 items that measure the ability to adapt one’s level of control according to the situation that they are confronted with. Responses range from *Does not apply at all* to *Applies very strongly* and are scored on a 4-point scale. This ERS has demonstrated acceptable internal consistency and positive associations with measures of well-being (Ndetei et al., 2019). We could not find any previous application of the ERS in South African research.

The SOC-13 comprises 13 items that assess the ability to identify and utilize one’s intrinsic and extrinsic resources to successfully negotiate stressors and maintain health. This scale comprises three subscales: meaningfulness (four items), comprehensibility (five items), and manageability (four items). In this scale, “Do you have the feeling that you are in an unfamiliar situation and don’t know what to do?” and “How often do you have the feeling that there’s little meaning in the things you do in your daily life?” are the examples of the items included. Respondents are required to indicate whether they agree or disagree in respect of each item. The reliability estimates for this scale have been reported to range from α = .70 to .92 (Paika et al., 2017). Strümpfer and Bands (1996) reported an α coefficient of .83 for the SOC-13 when used with a sample of South African Anglican priests.

### Procedure

An Internet-based survey was created and disseminated to a random sample of South African students during the period March 2020 to June 2020, which coincided with the strict national lockdown in the country.

### Ethical considerations

The university’s research ethics committee granted ethics clearance for the project (ethics reference no. HS20/5/1). Participants provided informed consent and completed the survey anonymously. No incentives were offered for participation.

### Data analysis

IBM SPSS Statistics for Windows (version 26; IBM Corp., Armonk, NY, USA) was used to determine descriptive statistics, intercorrelations, and the reliabilities of the scales. Path analysis with IBM SPSS Amos (version 26; IBM Corp.) was used to determine the direct and indirect effects of depression and hopelessness. Amos also provides bootstrapped confidence intervals and associated *p*-values which are used to determine the significance of the direct and indirect effects. The direct and indirect effects are said to be significant if zero does not fall within the confidence interval (Kenny, 2018). The user-defined estimands function of Amos was used to determine the mediating effects of each mediator separately.

## Results

Table 1 presents the descriptive statistics, intercorrelations, and reliabilities (α coefficient) for the variables used in the study.

**Table 1.** Intercorrelations, descriptive statistics, and reliabilities of variables.

Variable	1	2	3	4	5
1. Depression	–				
2. Hopelessness	.56***	–			
3. Satisfaction	−.51***	−.58***	–		
4. Sense of coherence	−.71***	−.57***	.54***	–	
5. Ego-resilience	−.42***	−.48***	.45***		
*M*	27.5	4.7	20.0	50.1	41.4
*SD*	13.4	4.4	7.7	12.7	6.8
α	.92	.88	.89	.81	.82

*** *p* < .001.

Mean scores for depression (*M* = 27.5 ± 13.4) and loneliness (*M* = 4.7 ± 4.4) in this study were found to be greater than those reported in the previous research (e.g., depression: Ramos et al., 2019, *M* = 19.8 ± 10.9; hopelessness: Aloba et al., 2017, *M* = 4.0 ± 4.3). Using the cutoff scores suggested in the literature (depression: Yoon et al., 2018; hopelessness: Aloba et al., 2017), we also found that 24.6% of the sample can be considered to be moderately depressed (CES-D score: >16); 54% can be considered to be severely depressed (CES-D score: >25); and 30.3% (BHS score: 4–8), 11.6% (BHS score: 9–14), and 5% (BHS score: 15–20) can be considered to have mild, moderate, and severe levels of hopelessness, respectively. With respect to satisfaction with life, the participants reported lower levels of life satisfaction (*M* = 20.0 ± 7.7) compared to the existing literature (e.g., Hinz et al., 2018; *M* = 26.5 ± 5.5).

There were no significant gender differences in terms of hopelessness (*t* = .335, *p* = .729), life satisfaction (*t* = .58, *p* = .574), ego-resilience (*t* = 1.36, *p* = .176), and sense of coherence (*t* = 1.67, *p* = .114). Women, however, reported higher levels of depression (*M* = 28.5, *SD* = 13.2, *t* = 3.03, *p* = .003) than men (*M* = 23.3, *SD* = 12.9).

In terms of the intercorrelations, Table 1 indicates that the indices of psychological distress correlated positively with each other (*r*_335_ = .56, *p* < .001) and negatively with life satisfaction (depression: *r*_335_ = −.51, *p* < .001; hopelessness: *r*_335_ = −.58, *p* < .001), as well as with those variables that are assumed to have a protective function, namely, sense of coherence (depression: *r*_335_ = −.71, *p* < .001; hopelessness: *r*_335_ = −.57, *p* < .001) and ego-resilience (depression: *r*_335_ = −.42, *p* < .001; hopelessness: *r*_335_ = −.48, *p* < .001). Finally, sense of coherence (*r*_335_ = .54, *p* < .001) and ego-resilience (*r*_335_ = .45, *p* < .001) were positively correlated with life satisfaction.

Table 1 further indicates that the various scales can be considered highly reliable. The α coefficient for the scales was found to range between .81 and .92, which is comparable to previously reported reliabilities for these scales (CES-D: Dol et al., 2020; BHS: Aloba et al., 2017; ER89: Chen et al., 2020; SOC-13: Vatandaşlar et al., 2020; SWLS: Hinz et al., 2018).

**Figure 1.**
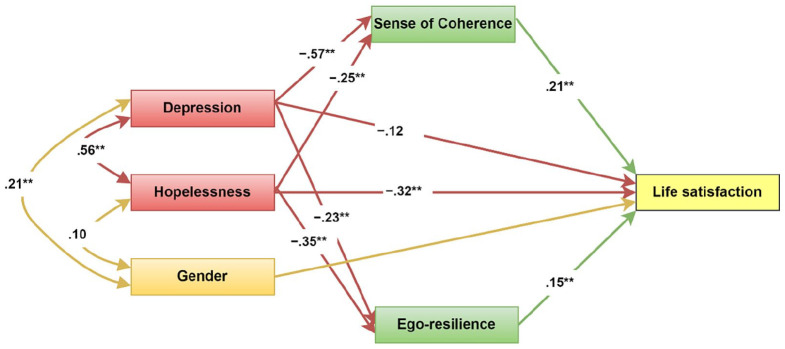
Structural equation model of the mediating role of sense of coherence and ego-resilience. Standardized effects presented. **p < .001.

Figure 1 shows the structural equation model tested. In this model, depression and hopelessness were used as the predictors, life satisfaction was used as an outcome, and sense of coherence and ego-resilience were used as the mediators. Given the significant gender differences in reported depression, gender was added to the structural equation model as a covariate.

In the absence of the mediator, there was a significant direct association between hopelessness (β = −.57, *p* < .001) as well as depression (β = −.51, *p* < .001). According to Table 2, in the presence of the mediator, the association between hopelessness and life satisfaction was still significant (β = −.32, *p* = .002) but reduced, whereas the association between depression and life satisfaction was no longer significant (β = −.12, *p* = .075). The direct effects of sense of coherence (β = .21, *p* = .003) and ego-resilience (β = .15, *p* = .003) on life satisfaction were also significant. Furthermore, the total indirect effects of depression (β = −.16, *p* = .001) and hopelessness (β = −.10, *p* = .001) on satisfaction were significant. This points to the joint mediating role of ego-resilience and sense of coherence.

**Table 2.** Direct and indirect effects of the study variables.

Variable	β	95% CI	β	*p*
Direct effects				
Hopelessness → satisfaction	−.551	[−.401, −.233]	−.319	.001
Depression → satisfaction	−.068	[−.226, .010]	−.118	.078
Sense of coherence → satisfaction	.129	[.101, .317]	.213	.001
Ego-resilience → satisfaction	.169	[.069, .234]	.149	.001
Hopelessness → sense of coherence	−.704	[−.322, −.166]	−.246	.001
Hopelessness → ego-resilience	−.529	[−.429, −.251]	−.347	.001
Depression → sense of coherence	−.542	[−.632, −.503]	−.572	.001
Depression → ego-resilience	−.115	[−.316, −.140]	−.228	.001
Total indirect effects				
Hopelessness → satisfaction^a^	−.180	[−.257, −.116]	−.104	.001
Depression → satisfaction^b^	−.089	[−.129, −.051]	−.156	.001
Specific indirect effects				
Hopelessness → ego → satisfaction	−.089	[−.149, −.044]	−.052	.001
Hopelessness → SOC → satisfaction	−.090	[−.157, −.045]	−.052	.000
Depression → Ego → satisfaction	−.019	[−.037, −.009]	−.034	.001
Depression → SOC → satisfaction	−.070	[−.107, −.033]	−.121	.001

CI: confidence interval; ego: ego-resilience; SOC: sense of coherence.

a Hopelessness as a predictor with sense of coherence and ego-resilience as the joint mediators.

b Depression as a predictor with sense of coherence and ego-resilience as the joint mediators.

Furthermore, all of the specific indirect effects were significant, namely, hopelessness to satisfaction through ego-resilience (β = −.05, *p* = .002) and through sense of coherence (β = −.05, *p* = .001), as well as depression to satisfaction through ego-resilience (β = −.03, *p* = .002) and through sense of coherence (β = −.12, *p* = .001). This indicates that sense of coherence and ego-resilience also independently mediate the hopelessness–life satisfaction relationship and depression–life satisfaction relationship. Since the direct effects of depression on satisfaction were not significant while the indirect effects through ego-resilience and sense of coherence were, this indicates that these relationships are fully mediated by ego-resilience and sense of coherence. In respect of the hopelessness–life satisfaction relationship, the results demonstrate a partial mediating role for ego-resilience and sense of coherence.

## Discussion

In this study, we investigated the impact of the COVID-19 pandemic on the psychological well-being of South African students and the potential protective role of ego-resilience and sense of coherence. First, compared to the results in the literature, we found greater levels of loneliness, depression, and hopelessness among the current sample, which were related to reduced satisfaction with life (Aloba et al., 2017; Ramos et al., 2019). This finding was expected and can be ascribed to the disruption of academic routines, challenges in adapting to remote online learning, fears of being infected with the virus, and uncertainty about the trajectory of the pandemic (Padmanabhanunni & Pretorius, 2021). Most students registered at the university are from working-class families, and the pandemic-related restrictions may have had implications for their parents’ job security and family income, which may lead to distress and reduce life satisfaction. In addition, limited access to friends, peers, and university-related resources (e.g., supportive staff, counseling facilities) may have led to greater levels of depression, loneliness, and hopelessness (Naidoo & Cartwright, 2020).

Second, the association between hopelessness and satisfaction with life was found to be only partially mediated by ego-resilience and sense of coherence, whereas the depression–life satisfaction relationship was found to be fully mediated by these protective factors. This confirms prior research findings (Gómez-Salgado et al., 2020; Y. J. Kim et al., 2015; Schäfer et al., 2020) and proposes that people with a greater sense of coherence and ego-resilience are better able to manage psychological stressors. According to Beck et al. (1974), depressive symptoms and hopelessness can arise from a particular attributional style characterized by negative cognitive appraisals about the self, significant others, and the future. This style is also characterized by appraisals that no responses in the individual’s repertoire will prevent adverse outcomes from occurring (Beck et al., 1974). Hence, in the context of COVID-19, it is possible that those with such an attributional style may be more vulnerable to viewing their situation as enduring and believe that they have limited power to effect meaningful change, which may, in turn, impact their life satisfaction. It is also plausible that individuals with greater ego-resilience and sense of coherence are less inclined toward such negative appraisals and better able to access resources for coping (Cole et al., 2015).

Finally, ego-resilience and sense of coherence had direct effects on satisfaction with life, indicating that these personality characteristics are health-sustaining ones. This confirms prior research on the association between low ego-resilience as well as low sense of coherence and student distress (e.g., Cole et al., 2015; Shankland et al., 2019). These findings have important implications for intervention. Existing research has suggested (e.g., Davidson et al., 2012) shown that group interventions focused on identifying internal and external resources for coping and enhancing awareness of these resources have a beneficial impact on self-efficacy and sense of coherence. Problem-focused coping strategies and mindfulness-based interventions can promote adaptation and ego-resilience by reorienting students to view stressors as motivating challenges rather than as enduring problems (Shankland et al., 2019).

The study was cross-sectional in nature, which limits inferences around causality. Therefore, longitudinal research is necessary to more conclusively assess the parallel mediating roles of ego-resilience and sense of coherence. This study also relied solely on self-report data, which may be affected by the tendency to only endorse favorable attitudes. In addition, only undergraduate students took part in the study. Hence, future research with more diverse samples can help confirm the results.

## Conclusion

Although this study is largely exploratory in nature, it provides important insights into the role of factors that are presumed to protect mental health, such as ego-resilience and sense of coherence. It also suggests potential pathways between these protective factors and indices of psychological well-being.
